# Transforming Pediatric Dentistry with Chitosan Gel: Current Evidence and Emerging Trends—A Scoping Review

**DOI:** 10.3390/ma19183951

**Published:** 2026-09-17

**Authors:** Dhruvi Raj Solanki, Punit Fulzele, Ishani Rahate, Aakriti Chandra, Anjori Raut

**Affiliations:** Department of Pediatric and Preventive Dentistry, Sharad Pawar Dental College, Datta Meghe Institute of Higher Education and Research, Sawangi (Meghe), Wardha 442001, Maharashtra, India; punitr007@gmail.com (P.F.);

**Keywords:** chitosan, pediatric dentistry, remineralizing agent, dental caries, pulp remineralization, dental materials, antibacterial agents, pulpotomy, gel, mouthwash

## Abstract

Chitosan, a hydrogel derived from chitin via deacetylation, exhibits antimicrobial, bioadhesive, and regenerative properties, making it a promising biomaterial in pediatric dentistry. Despite extensive research, a comprehensive review of its preventive, restorative, and regenerative applications in children is lacking. This review consolidates current evidence on the mechanisms, clinical uses, and future potential of chitosan-based interventions in pediatric dental care. The search identified a broad spectrum of evidence encompassing preventive, restorative, and endodontic uses of chitosan. Chitosan-based mouthwashes, varnishes, and remineralizing agents demonstrated significant reductions antimicrobial and remineralizing effects, primarily in in vitro studies and limited clinical trials. Incorporating chitosan into cements, adhesives, and resins improved fluoride release, durability, and antibacterial activity without compromising mechanical strength as demonstrated in lab-based studies. In pulp therapy, chitosan nanoparticles enhanced intracanal medicaments, supported hemostasis in pulpotomy, and promoted odontogenic differentiation in regenerative procedures. Although current in vitro and early clinical data are encouraging and present chitosan as a promising biomaterial in the field of pediatric dentistry, large, well-designed trials and standardized formulations are essential to validate long-term safety, and enable consistent integration of chitosan gel-based materials into routine pediatric dental care.

## 1. Introduction

**Figure 1:**
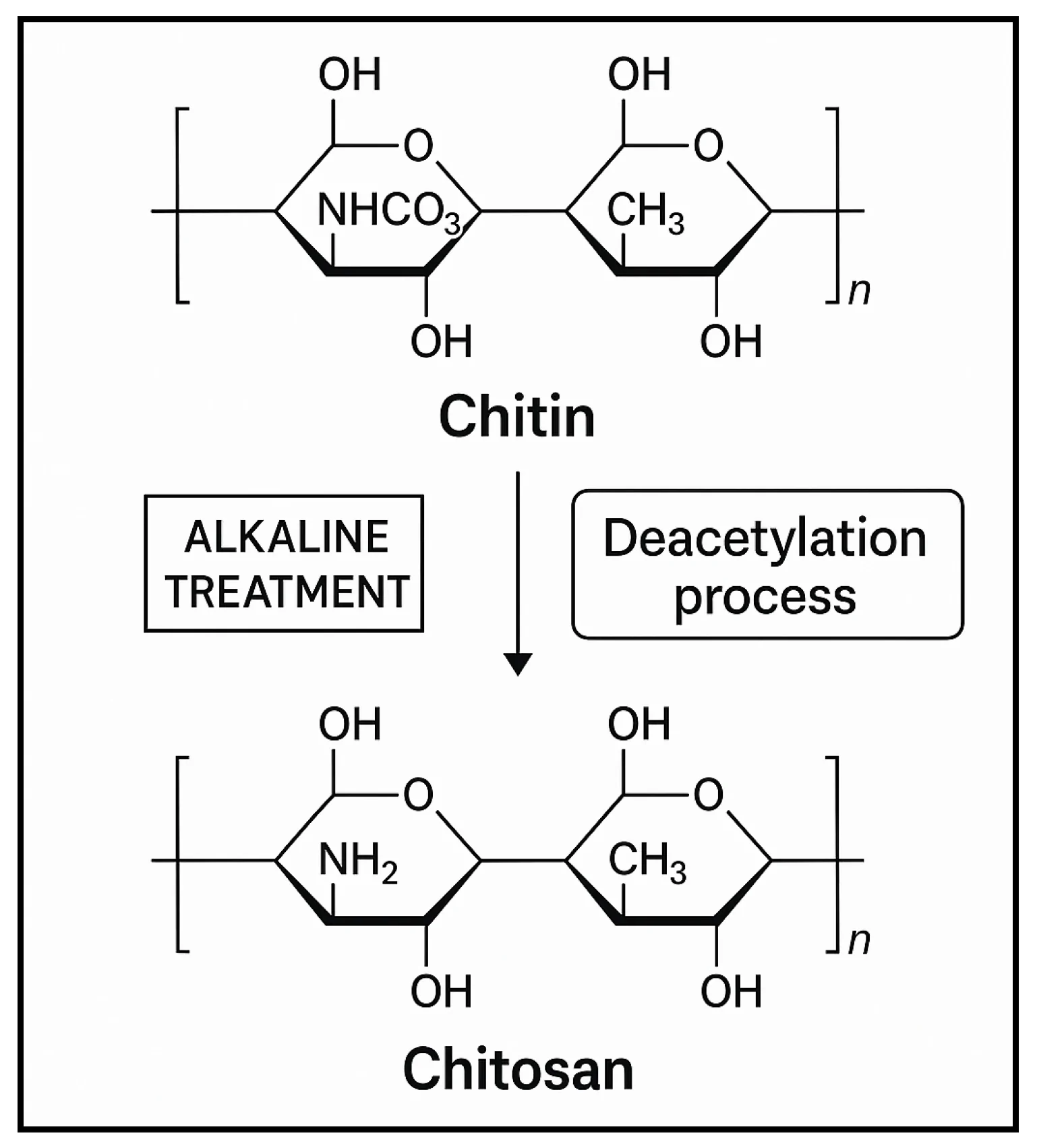
Deacetylation of chitin and formation of chitosan.

Gels have garnered significant interest in recent years, with marked progress in developing safe and functional materials for biomedical use. Whether natural or synthetic, these materials are specifically designed for implantation within biological systems. Chitin, a natural biomaterial with numerous biomedical uses, is derived from both plants and animals. Chitin, the second most abundant natural polymer on Earth, is primarily derived from marine shells. It has a linear structure with a molecular weight (MW) exceeding several thousand kiloDaltons (kDa) and is composed of β-(1→4)-linked N-acetyl-D-glucosamine units [1,2]. A unique feature of chitin, distinguishing it from most polysaccharides, is its nitrogen content present as amino groups, alongside carbon, oxygen, and hydrogen (Figure 1). Despite its notable properties, chitin’s hydrophobicity limits its applications. Consequently, attention has shifted to chitosan, a hydrogel and derivative obtained by deacetylation of chitin [3,4]. Chitosan gel (CS), an emerging polymer in dentistry, has been widely studied and recognized for its anti-caries potential since its discovery by Rouget in 1859. It is an FDA-certified cationic copolymer composed of β-(1-4)-linked 2-amino-2-deoxy-D-glucose and 2-acetamino-2-deoxy-D-glucoside units. Its MW generally ranges from 10 to 1000 kDa, which also influences its biological activity [5,6,7]. The quality of chitosan depends on the source of chitin and the method of extraction. Degree of deacetylation (DD) governs chitosan’s physical, chemical, and biological properties by determining the free amino groups exposed and its resulting molecular weight [8]. Solubility of CS depends on pH, molecular weight, and DD%—higher molecular weight reduces solubility due to hydrogen bonding, while higher DD increases solubility through amino group protonation. CS is classified as low (55–70% DD, largely insoluble), medium (70–85%, partly soluble), high (85–95%, highly soluble), or ultrahigh (95–100%, rarely achieved) based on the deacetylation degree, with higher DD generally yielding better solubility in acid [9].

**Figure 2:**
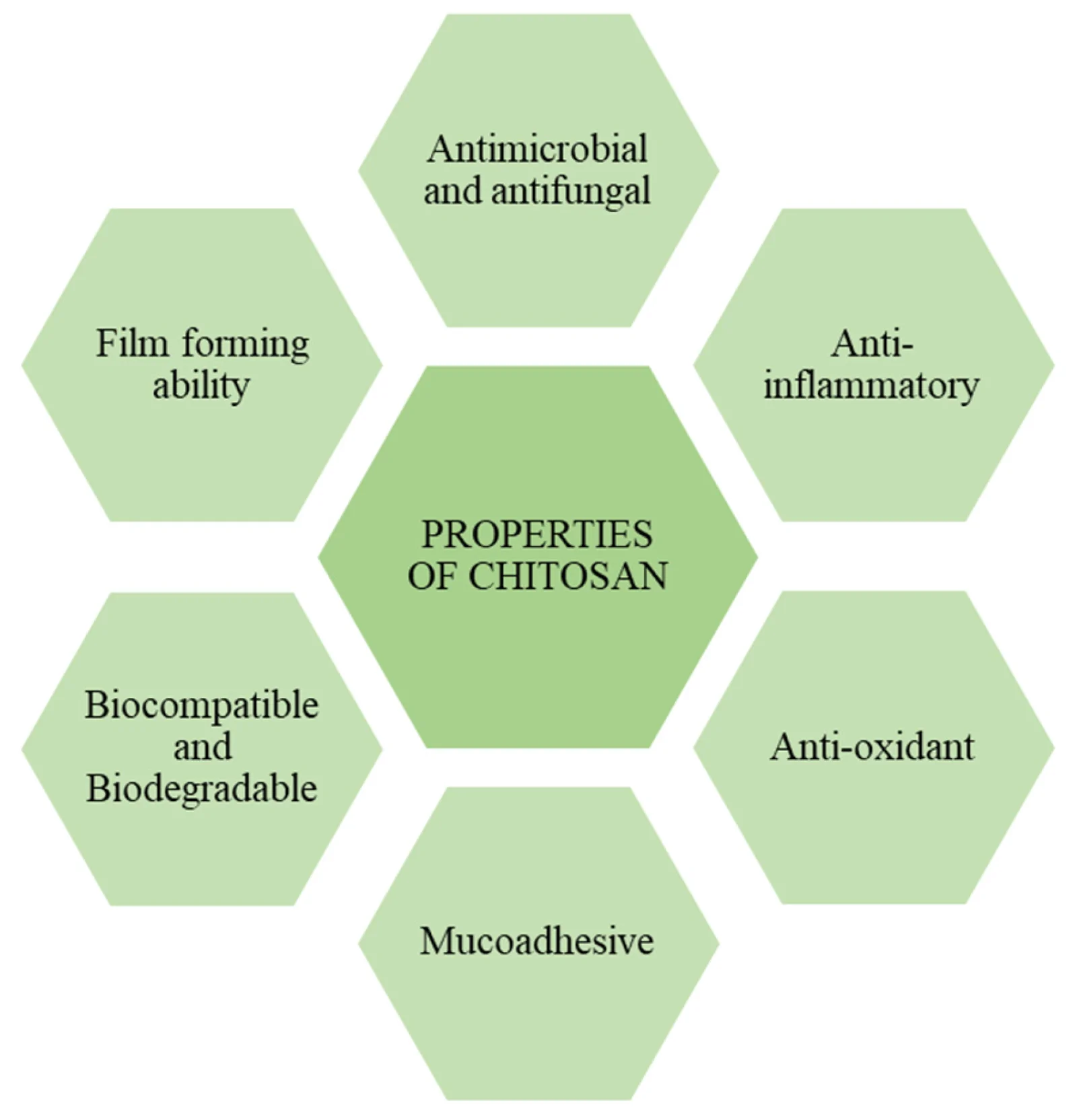
Properties of chitosan.

**Figure 3:**
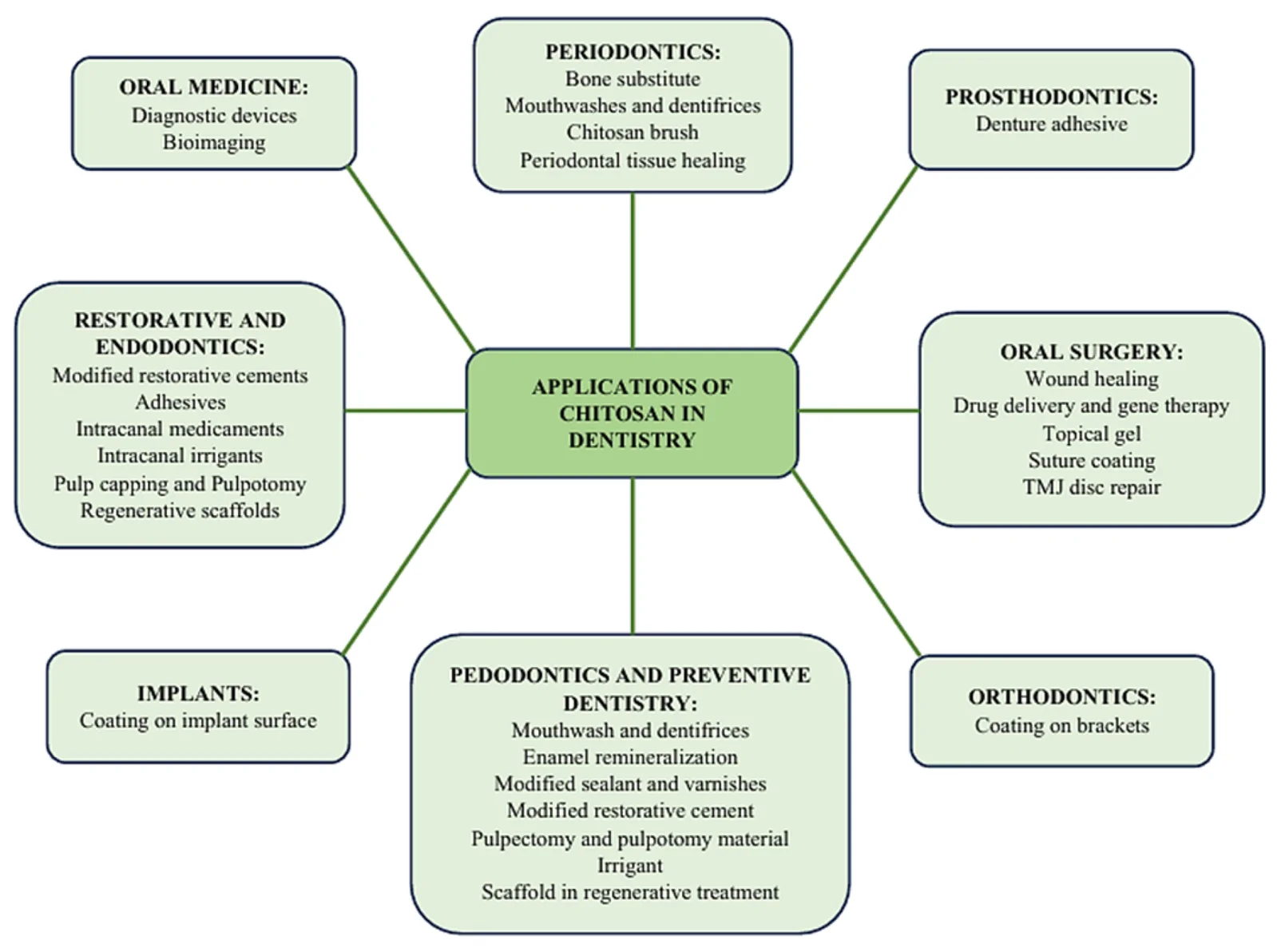
Applications of chitosan in dentistry.

Owing to its antimicrobial, biocompatible, bioadhesive, hemostatic, and regenerative properties, among others (Figure 2), CS has been explored in preventive dentistry, pediatric dentistry, restorative dentistry, periodontics, endodontics, prosthodontics, orthodontics, oral medicine, oral surgery, and implantology (Figure 3). Its versatility enables applications ranging from caries prevention and restorative material modification to wound healing, tissue regeneration, implant surface coatings, and controlled drug delivery, highlighting its potential as a multifunctional biomaterial in contemporary dental practice [10]. It supports the development of preventive products and clinical adjuncts, including biomimetic mineralization, dentin adhesion, and targeted drug delivery systems. These advancements highlight its significant potential for diverse applications in the field [11,12,13,14].

Pediatric dentistry usually deals with infants, children and adolescents, thereby addressing both primary and permanent dentition. It presents distinct clinical challenges that necessitate the use of biomaterials that are biocompatible, resorbable, minimally invasive, and capable of supporting the preservation of hard and soft tissue. The scope of pediatric dentistry encompasses not only therapeutic aspects (restoration and endodontic treatment) but also preventive aspects and regenerative procedures for young permanent teeth [15]. Natural products such as herbal extracts, propolis, fumaric acid, chitin, and CS have gained attention because of their biocompatibility, antimicrobial activity, low toxicity, and potential to support tissue regeneration. Organic formulations incorporating plant-derived ingredients and biopolymers are increasingly being explored as alternatives to conventional synthetic products [16]. Within pediatric dentistry, the clinical applicability of CS gel can be organized across three major axes: preventive, restorative, and endodontic–regenerative domains. The preventive axis focuses on the remineralization of incipient lesions and prevention of demineralization to halt caries progression and prevent new lesions. The antimicrobial action, biofilm inhibition, and drug delivery property of CS help achieve this [17]. The restorative axis involves the removal of decayed tooth structure and subsequent restoration to maintain its structural integrity and function [18]. This is achieved by combining CS with restorative materials to enhance their properties. The endodontic and regenerative axis includes applications in intracanal medicaments, pulp capping, pulpotomy, and regenerative procedures, in which CS’s properties contribute to the preservation of pulp vitality and dentin–pulp complex regeneration [19].

The literature lacks a detailed compilation of the applications of CS gel in pediatric dentistry. This review aims to bridge this knowledge gap and provide a comprehensive overview of CS gel applications in pediatric dentistry by compiling the evidence on the uses of CS gel in pediatric dentistry. Understanding CS’s potential could enhance its use in various treatments in pediatric dentistry, including preventive, restorative, endodontic, and pulp regeneration therapy. By highlighting its mechanisms of action, clinical relevance, and prospects, this review seeks to provide a coherent scientific basis for the potential integration of CS gel-based materials into routine pediatric dental practice.

## 2. Materials and Methods

Research was gathered focusing on the information on the diverse applications, advantages and mechanisms of CS from PubMed, Scopus and grey literature using the following search string: (chitosan*[Title/Abstract]) OR (chitosan[MeSH Terms]) AND (teeth[Title/Abstract]) OR (tooth[Title/Abstract]) OR (“pediatric dentistry”[Title/Abstract]) OR (“paediatric dentistry”[Title/Abstract]) OR (dentistry, pediatric[MeSH Terms]) AND (((((((((((((((((((((((((((((((((((((dental caries[MeSH Terms]) OR (tooth demineralization[MeSH Terms])) OR (tooth remineralization[MeSH Terms])) OR (dental plaque[MeSH Terms])) OR (application, fluoride[MeSH Terms])) OR (mouthwashes[MeSH Terms])) OR (bonding agent, dentin[MeSH Terms])) OR (agent, dentin bonding[MeSH Terms])) OR (dental sealant[MeSH Terms])) OR (dental cement[MeSH Terms])) OR (dental bonding[MeSH Terms])) OR (pulpotomies[MeSH Terms])) OR (pulpotomy[MeSH Terms])) OR (pulpectomy[MeSH Terms])) OR (pulpectomies[MeSH Terms])) OR (pulp capping and pulpectomy agents[MeSH Terms])) OR (agent, pulp capping[MeSH Terms])) OR (agents, pulp capping[MeSH Terms])) OR (restor*[Title/Abstract])) OR (demineral*[Title/Abstract])) OR (remineral*[Title/Abstract])) OR (fluoride*[Title/Abstract])) OR (caries[Title/Abstract])) OR (“pulp capping”[Title/Abstract])) OR (pulpo*[Title/Abstract])) OR (pulpec*[Title/Abstract])) OR (regenrat*[Title/Abstract])) OR (prevent*[Title/Abstract])) OR (sealant[Title/Abstract])) OR (varnish[Title/Abstract])) OR (gel[Title/Abstract])) OR (dentifrice*[Title/Abstract])) OR (toothpaste[Title/Abstract])) OR (cement[Title/Abstract])) OR (composite[Title/Abstract])) OR (“bonding agent”[Title/Abstract])) OR (irriga*[Title/Abstract])) OR (“root canal”[Title/Abstract]).

**Figure 4:**
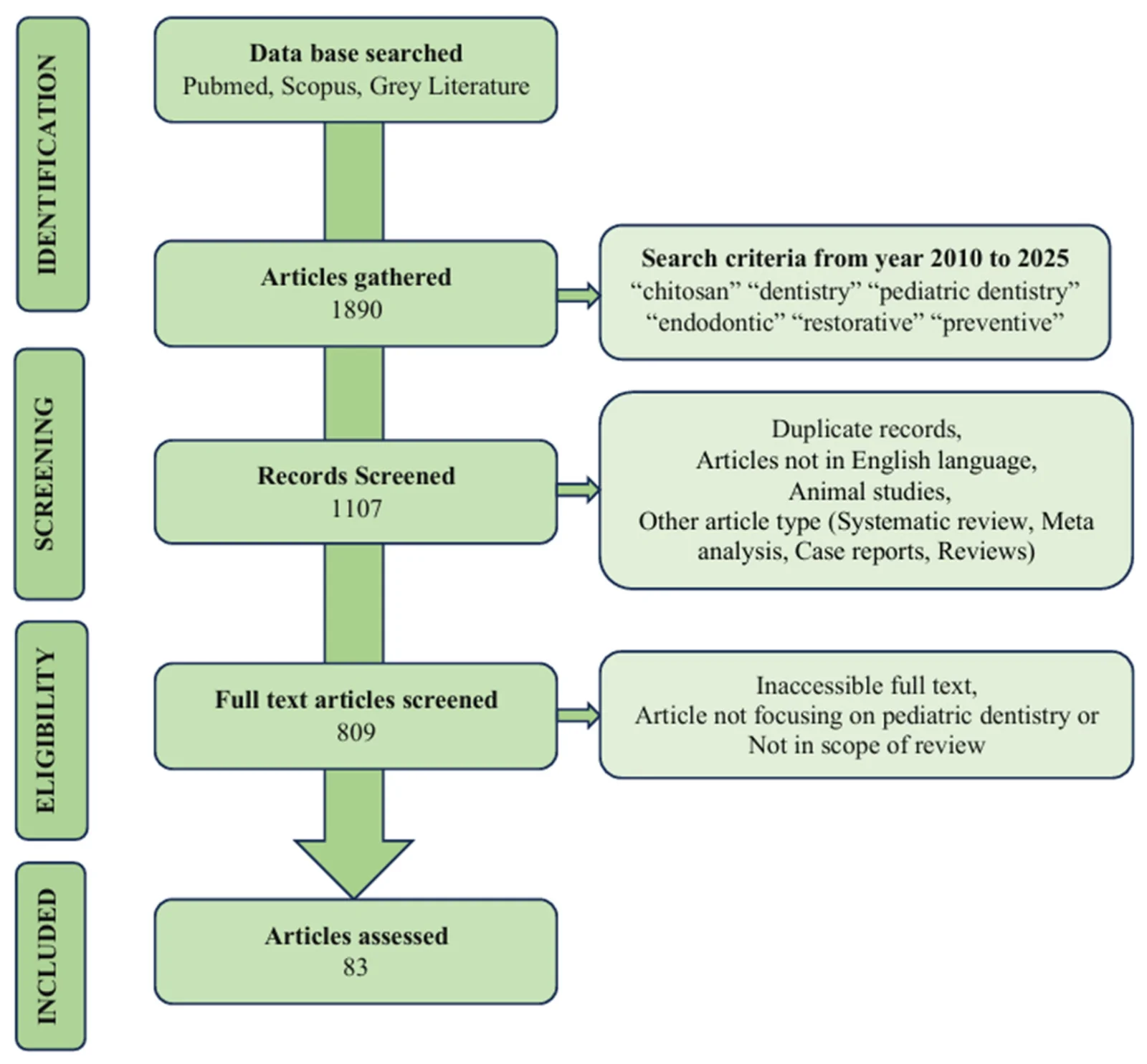
Article search strategy.

The initial search across PubMed, Scopus, and grey literature identified 1890 records published between 2010 and 2025 as per the search criteria. After the removal of 783 duplicate records, 1107 unique articles remained. These records were further screened, resulting in the exclusion of 298 articles. The primary reasons for exclusion were animal studies (n = 126), non-English-language publications (n = 69), and review articles, meta-analyses, or case reports (n = 103). The remaining 809 articles were assessed for full-text eligibility. Of these, 726 articles were excluded due to inaccessible full text (n = 129) or lack of relevance to chitosan use in pediatric dental applications, including caries prevention, restorative dentistry, endodontics, or regenerative procedures (n = 597). Ultimately, 83 studies met the inclusion criteria and were included in the qualitative synthesis (Figure 4). The review was designed in accordance with the PRISMA guidelines (“see Supplementary Materials”) and registered on OSF registries as “Transforming Pediatric Dentistry with Chitosan Gel: Current Evidence and Emerging Trends—a Scoping Review” (https://osf.io/tgex5).

## 3. Results and Discussion

### 3.1. Mechanism of Action

#### 3.1.1. Antibacterial Mechanism of Chitosan

Low-MW CS exhibits greater bacterial binding flexibility, penetrating membranes, disrupting DNA, inhibiting macromolecule synthesis, and interfering with *Streptococcus mutans* (*S. mutans*) polysaccharide synthesis (by inhibiting the activity of dextransucrase, an essential enzyme in the *S. mutans*) and virulence factors, leading to bacterial inactivation in microbial studies [20,21,22,23]. A high degree of deacetylation in CS enhances its positive charge, improving bacterial adherence and bactericidal effects [21]. However, contrary to previous findings, high-MW CS demonstrated lower minimum inhibitory and bactericidal concentrations against *S. mutans*, indicating superior anti-caries efficacy. Its strong positive charge facilitates bacterial membrane disruption, enhancing its antimicrobial properties as demonstrated in in vitro studies [24,25]. CS’s applications are limited by its low solubility in water and organic solvents, though its amino groups allow for various chemical modifications that improve its physical and chemical characteristics, solubility, antibacterial effects, and drug absorption and provide chelating capabilities. Thus, both MW and the degree of deacetylation are crucial for CS’s antibacterial efficacy and applications, along with multiple variables like formulation, concentration and status of oral ecology.

The precise antibacterial mechanism remains unclear, as variations in microbial surface charge and the complexity of the oral microbiome influence its efficacy [26,27]. Human studies need to be conducted to translate the in vitro results into clinical settings.

#### 3.1.2. pH Response Property of Chitosan

Sugars in dental plaque lower pH, promoting tooth demineralization, while sustained acidity prolongs mineral loss [28]. When pH drops below the critical threshold, apatite dissolves, releasing calcium and phosphate ions, that can redeposit during pH recovery, aiding in dental repair. Low pH hinders mineralization. CS, a cationic polymer, is protonated in acidic conditions, enhancing its solubility and antibacterial efficacy [29]. This pH-responsive behavior makes CS effective in caries prevention. Boda SK has developed pH-responsive CS nanomaterials that release antibacterial agents in acidic environments, targeting pathogenic plaque. Protonation enhances CS’s positive charge, improving its adherence to negatively charged bacterial surfaces, thereby enhancing its antibacterial effect in acidic conditions [30]. Additionally, CS’s charged amino groups capture hydrogen ions, forming a protective layer that buffers pH fluctuations, preventing further demineralization. As pH normalizes, CS dissociates, facilitating enamel remineralization [31].

### 3.2. Applications in Pediatric Dentistry

#### 3.2.1. Oral Hygiene

CS’s antimicrobial properties have led to its use in oral health products to inhibit plaque formation. It has been used in various forms like mouthwash, dentifrice, chewing gum, sealant, and remineralizing agent, etc. In vitro studies and a few early clinical trial studies show that CS mouthwash is highly effective, achieving 99.99% antibacterial activity within 20 s, comparable to or surpassing other commercial mouthwashes [32]. It prevents microbial attachment and biofilm formation by *Prevotella intermedia*, *Candida albicans*, *S. mutans*, and Enterococcus faecalis, thereby preventing caries [33,34,35]. CS’s antibacterial efficacy can be enhanced by combining it with photodynamic therapy, as shown in an in vitro study, allowing CS to penetrate bacterial biofilms and deliver photosensitizers as well as other materials [36,37,38,39].

#### 3.2.2. Preventive Therapy

- Chitosan-aided mineralization of enamel and dentin

In vitro studies of CS show significant promise in biomimetic mineralization due to its functional group’s ability to induce apatite nucleation [40]. It forms a protective barrier, inhibiting enamel demineralization and preventing mineral ion release by blocking organic acids [41]. Its positive charge enhances adhesion to negatively charged demineralized enamel, while carboxyl and hydroxyl groups in CS chelate calcium ions, stabilizing amorphous calcium phosphate (ACP), preventing enamel precipitation and facilitating ion diffusion into deeper enamel and dentinal lesions and aiding calcium phosphate nucleation [42,43,44,45]. Leveraging these properties, researchers have developed CS-based remineralizing agents in vitro, such as CS/calcium phosphate hybrid microgel [46].

In dentin, the selective permeability of type I collagen allows infiltration of ACP while excluding high-molecular-weight carboxymethyl CS, promoting intrafibrillar mineralization and improving mechanical properties while preventing premature crystallization [47,48]. Additionally, it also protects dentinal collagen by forming cross-links, blocking collagenase binding sites, and directly inhibiting collagenase activity as demonstrated in experimental studies [49]. Although in vitro studies consistently demonstrate the ability of CS-based systems to promote remineralization, these outcomes should be interpreted as proof-of-concept rather than clinical efficacy.

- Chitosan combined with fluoride

Fluorides (F) used professionally or at home are effective in caries reduction [50]. Recent studies show that biopolymers like CS, alginate, and pectin can form stable, bioadhesive nanoparticles for F delivery, with CS being the most efficient carrier. This constant delivery system enhances caries protection, as supported by experimental studies and limited clinical trials [51,52,53]. Microparticles incorporating CS as a carrier for sodium fluoride also show potential in improving F ion release and enhancing preventive action. The slow-releasing F CS-coated nanoparticles not only deliver F directly to enamel but also increase F content on the tooth surface, thereby enhancing resistance to decay [54].

In an in vitro study, Targino employed CS as a delivery medium for silver nanoparticles and F. The study demonstrated that CS exhibited low toxicity and high efficacy at low concentrations, surpassing chlorhexidine (CHX). Both silver and F, along with CS, demonstrated antimicrobial activity against *S. mutans*, indicating potential to control tooth decay and reducing dental caries [55]. Similarly, dos Santos et al. also utilized CS as an agent for nanosilver fluoride in a clinical trial [56]. More clinical trials on nanoparticle-based caries prevention for children are required to provide strong evidence.

- Other preventive agents

Ruan et al. assessed the use of amelogenin–CS nanoparticles within a hydrogel in vitro for enamel remineralization. Their findings revealed that the hydrogel notably enhanced the mechanical properties of the treated enamel while effectively inhibiting bacterial growth. Thus, it provided two mechanisms for caries prevention: mineralization through apatite crystal regrowth and inhibition of dental biofilm accumulation [57]. Different forms of CS combinations used for preventive applications are listed in Table 1.

- Caries vaccine

CS enhances mucosal delivery and antigen transfection for anti-caries vaccines, aiding both drug transport and vaccine delivery. Its positive charge protects deoxyribonucleic acid and facilitates absorption by forming nanocomposites, thereby improving its antigen transfection efficiency [47]. Novel nanoparticles combining CS with anionic liposomes offer pH-responsive release and facilitating nasal delivery. CS also acts as an immunostimulatory adjuvant, boosting immune responses. Bi et al. (2019) [58] used CS combined with two adjuvants—Pam3CSK4 (TLR2 agonist) and monophosphoryl lipid A (MPL, TLR4 agonist)—to enhance the immune response to recombinant *S. mutans* Pac protein. Their study found that these CS-adjuvanted formulations significantly increased Pac-specific antibody levels in serum and saliva, thereby reducing the severity of dental caries [58]. However, current evidence is confined to in vitro and animal studies, and no clinical data are available to support their use in caries prevention in children. Concerns about its potential cytotoxicity due to its positive charge warrant further investigation into its safety and efficacy, and also, clinical trials need to be conducted [59].

**Table 1:** Summary of studies on preventive applications of chitosan in pediatric dentistry.

S. No	Preventive Application	Author (Year)	Type of Study	Comparison Groups	Test Parameters	Result	Strength of Evidence
1	Dentifrice	Francese MM et al., 2024 [60]	Randomized controlled trial (RCT)	Titanium F/CS, Elmex, and Placebo	Erosive tooth wear	No significant difference.	Clinical (RCT)
2		Demir S, 2021 [61]	In vivo	Conventional toothpaste and toothpastes containing theobromine, aloe vera, miswak, propolis, CS, enzymes and probiotics, respectively	Antimicrobial efficacy	Theobromine, CS, and traditional toothpaste exhibited antimicrobial efficacy against all tested bacteria.	Clinical
3		Heryumani Sulandjari JCP, 2019 [62]	RCT	Control and CS	Plaque accumulation	Reduced plaque accumulation in CS group compared to control.	Clinical (RCT)
4	Mouthwash	Rahayu DP, 2022 [51]	In vitro	CHX gluconate, Listerine^®^ total care, CS3H, CS fluoride (CS3H F), *N*-(2(2,6-diaminohexanamide)-CS (CS3H Lys), CS, CS3H Lys F	Prevention of acid demineralization	CS3H Lys F proved to be 1.6-times more efficient in preventing acid demineralization.	Pre-clinical
5		Vilasan A, 2020 [63]	RCT	0.2% CHX, 0.5% CS, CHX—CS combination group	Plaque accumulation	CS combined with CHX showed a significant reduction in plaque compared with CHX or CS alone.	Clinical (RCT)
6		Mhaske SP, 2018 [64]	Clinical trial	0.2% CHX digluconate, 2% CS solution, 0.2% CHX/2% CS combination	Plaque accumulation	CHX-CS group showed the lowest Plaque index, while Gingival Index was not significant.	Clinical
7	Remineralization	Wu L, 2018 [65]	In vitro	CS-ACP paste, control group and MI plus paste group	Remineralization	CS-ACP group had the highest remineralization width, followed by MI+ group.	Pre-clinical
8		Mohamed Y, 2023 [66]	In vitro	Sodium fluoride, silver diamine fluoride, phosphorylated CS nanoparticles (Pchi) and control	Remineralization	Enamel microhardness and remineralization potential were highest in Pchi and Silver diamine fluoride groups.	Pre-clinical
9		Zhang J, 2019 [67]	In vitro	Bioglass application on CS pre-treated lesions (CB), CS-bioglass slurry (CBS), standard remineralization solution, remineralization and deionized water	Subsurface remineralization	The CS-bioglass complex showed superior mineral regain and microhardness recovery.	Pre-clinical
10	Pit and fissure sealant (PFS)	Lai CC, 2022 [68]	In vitro	0%, 2%, 4% CS/fluoride microparticles (C/F), with ClinproTM fissure sealant	Antimicrobial efficacy	2% and 4% C/F reduced colony count ratios to 10% and 25%, respectively.	Pre-clinical
11		Hashemi Kamangar SS, 2021 [69]	In vitro	Master Dent fissure sealant alone and after incorporating CS nanoparticles	Antibacterial efficacy	1 weight% CS sealant reduced colony counts significantly.	Pre-clinical
12		Kritika Selvakumar, 2021 [70]	In vitro	Helioseal F, Helioseal + nanoCS Clinpro, Clinpro + nanoCS and Fuji VII glass ionomer cement (GIC), GIC + nanoCS	Wear resistance	Modified sealants showed marginally improved wear behavior.	Pre-clinical
13		Kumar Naina, 2025 [71]	RCT	Clinpro^TM^ and 2% CS-modified Clinpro^TM^ sealant	Sealant retention and caries incidence	CS-modified sealant demonstrated significantly superior retention and caries prevention.	Clinical (RCT)

#### 3.2.3. Restorative Therapy

Unlike adult restorative dentistry, pediatric restorative care is influenced by distinct anatomical, histological, and biological characteristics of primary and immature permanent teeth. Primary teeth exhibit thinner enamel and dentin, larger pulp chambers, higher organic content, and lower mineral density compared with permanent dentition, which predisposes them to rapid caries progression and early pulpal involvement [15,17,18,19]. Furthermore, primary dentin demonstrates greater tubular density and permeability, which may compromise hybrid layer formation and long-term adhesive stability. In addition, restorative strategies in children emphasize minimally invasive approaches, the preservation of pulp vitality, fluoride release, bioactivity, and reduced chairside time due to behavioral considerations [17,18,19]. A substantial amount of research and development has been undertaken to advance the field of dental restorative materials, with a particular focus on biomimetic approaches. These efforts include the designing and refinement of materials that closely mimic the natural properties of tooth structure, aiming to enhance both the functional and aesthetic outcomes of dental restorations (Table 2).

Recent advances have also explored the application of CS as bioactive surface coatings for restorative materials, implants, orthodontic appliances, and endodontic instruments. CS coatings tend to improve the surface characteristics, reduce bacterial adhesion, enhance tissue integration, and also serve as carriers. However, most studies are limited to in vitro settings [72].

- Modification of dental restorative materials

Glass ionomer cement (GIC) is frequently used for prosthesis cementation and restorations. Recent advances include incorporating resins or nano-additives for improved performance [7,73]. The anionic groups of polyacrylic acid in GIC interact with the cationic amine groups of CS, forming an interpenetrating polymer network, thereby enhancing adhesion [73]. Pre-clinical studies show that CS nanoparticle-enriched GIC increases F release and improves resistance to bending, wear, and pressure compared to traditional GIC. CS-modified GIC also shows no cytotoxic effects on pulp cells, making it suitable for bioactive dental restorations [74].

CS incorporation into composites lowers volumetric shrinkage and microleakage compared with conventional composites. Fourier Transform Infrared Spectroscopy (FTIR) analysis of CS-incorporated composite showed high reactivity of its amine groups in conjunction with the hydroxyl groups. Its high nitrogen content (6.89%) strengthens adhesion, reduces monomer leaching, and minimizes hydrolytic bond degradation [75].

- Adhesion and dentin bonding

Compared with permanent dentin, primary dentin contains a higher organic matrix content and exhibits reduced mineralization, which may increase susceptibility to collagen degradation and hydrolytic breakdown of the resin–dentin interface. Studies have demonstrated that CS can enhance collagen stabilization and reduce enzymatic degradation within demineralized dentin matrices [48,76]. The longevity and durability of restorations largely depend on the bond between resin adhesives and dentin. Challenges such as inadequate smear layer removal and the technique sensitivity of acid etching compromise tooth adhesion, thereby making the tooth susceptible to leakage [77]. The formation of covalent bonds between CS and dental resin and electrostatic interaction with demineralized dentin further enhance adhesion and hybrid layer stability [78,79].

In vitro studies have also shown that incorporating CS into “etch and rinse” adhesive systems enhances dentin permeability, bonding strength and restoration resistance. CS also reduces collagen breakdown and water permeation in hybrid layers, potentially aiding bacterial elimination [80]. CS’s antibacterial effect prevents bacterial infiltration into microgaps between restorative materials and dental tissues, thus preventing secondary caries [81].

- Limitations

Despite encouraging laboratory findings, inherent physicochemical barriers currently restrict the use of CS as a restorative biomaterial. Native CS exhibits relatively poor mechanical strength, limited wear resistance, high viscosity, and inadequate adhesive performance compared with contemporary resin-based composites and ceramic restorative materials. These physicochemical limitations may compromise long-term durability and clinical performance when used as a standalone restorative material. Consequently, current research primarily focuses on chemical modification of CS and its incorporation into hybrid biomaterials to improve its mechanical and adhesive properties while preserving its biological advantages [82,83].

Despite promising outcomes reported in laboratory and pre-clinical studies, the majority of evidence supporting CS-based modifications of restorative materials is derived from in vitro investigations. Clinical trials in pediatric populations are lacking to validate whether these favorable mechanical, antibacterial, and adhesive properties translate into predictable clinical performance. Also, the heterogeneity of the formulations is a concern for widespread acceptability and use.

**Table 2:** Summary of studies on restorative applications of chitosan in pediatric dentistry.

S. No	Restorative Application	Author (Year)	Type of Study	Comparison Groups	Test Parameters	Result	Strength of Evidence
1	Composite	Deb A, 2021 [75]	In vitro	Microhybrid composite, CS + composite	Microleakage	CS-composite group significantly decreased microleakage.	Pre-clinical
2		Tanaka C.B, 2020 [84]	In vitro	CS and CS/Dibasic calcium phosphate particles	Antimicrobial efficacy	All composites exhibited nearly 20% less biofilm than control.	Pre-clinical
3		Ali S, 2020 [85]	In vitro	Microhybrid and flowable resin bonded composites mixed with 0, 0.25, 0.5 and 1% *w*/*w* CS	Antibacterial and surface mechanical properties	No significant difference in antimicrobial property. Vicker’s hardness was significantly higher in 1% CS.	Pre-clinical
	GIC	Mishra A, 2017 [86]	In vitro	Conventional GIC, CH-modified GIC, CHX-CS-modified GIC	Antibacterial efficacy and physical properties	CH-modified GIC showed significant antimicrobial efficacy and higher flexural and compressive strength.	Pre-clinical
4		Nishanthine C et al., 2022 [87]	In vitro	NanoCS-modified type II light cure universal restorative, type II universal restorative, GC Fuji VII, and type IX	F-releasing	Greater F release demonstrated in CS-modified GICs.	Pre-clinical
5		Patel A, 2021 [88]	In vitro	Resin-modified GIC (RMGIC) and CS-infused RMGIC	Adhesive bond strength and F release	Incorporating CS into RMGIC does not affect its bond strength but increased F release.	Pre-clinical
6	Bioactive glass	Kazem N.E, 2024 [89]	In vitro	Control unmodified adhesive and 5 wt% bioactive glass-modified adhesive	Physical properties and microtensile bond strength	5 wt% Bioactive glass showed enhanced microtensile bond strength and bond durability.	Pre-clinical
7	Denting bonding	Ezz El-Din Y, 2023 [90]	In vitro	All-bond universal adhesive, control, CS (0.5), CS (1%), Nano-CS (0.5%) and Nano-CS (1%)	Wettability, pH and microtensile bond strength	Control and 0.5% CS groups had significantly higher contact angles. The control group had the highest pH, followed by Nano-CS (0.5%) and Nano-CS (1%). Nano-CS exhibited the highest bond strength.	Pre-clinical
8		Paschoini VL, 2021 [91]	In vitro	CS (control) or with 2.5% CS gel	Bond strength and durability	CS improved the microtensile bond strength of the adhesive interface in comparison to the control group.	Pre-clinical
9		Ziotti I.R, 2022 [92]	In vitro	Distilled water (control) and 2.5% CS solution	Strength	CS-treated specimens had higher bond strength than the untreated ones.	Pre-clinical

#### 3.2.4. Pulpal Therapy

The main objective of endodontic procedures is to reduce or alleviate microbial burden within the root canal system. To this end, several intracanal medications and irrigants have been developed and evaluated for their efficacy against both mono- and multi-species biofilms [93]. Incorporating CS nanoparticles into calcium hydroxide pastes enhances their efficacy as interappointment medications and improves the performance of endodontic sealers used for root canal filling [94] (Table 3).

- Pulp capping

Incorporating CS and its derivatives into resin-based materials enhances dentin bond strength and stability. In vitro studies have shown that carboxymethyl CS promotes dentin remineralization by releasing calcium and phosphate ions and improving monomer infiltration at the resin–dentin interface, making it a promising indirect pulp-capping agent [48,95]. Pre-treating the dentin bonding interface with high-MW CS significantly improves bond integrity, stabilizes internal minerals, protects collagen from protease activity, reduces water permeability of the resin–dentin hybrid layer, and exhibits antibacterial effects against bacterial biofilms [76].

Subhi et al., in a lab-based study, stated that gypsum-based CS material used as a pulp-capping agent significantly increased cell viability in pulp stem cells compared with Dycal. Additionally, using calcium phosphate carboxymethyl–CS composite as a pulp-capping material promoted the differentiation of pulp stem cells into odontoblastic cells. They also demonstrated rapid gelation, improved mechanical properties, biological compatibility, and odontogenic potential, making it a promising regenerative pulp-capping agent [96].

- Pulpotomy

CS is a potent hemostatic agent that forms a barrier by cross-linking with erythrocytes and platelets and boosting the production of platelet-derived growth factor-AB and transforming growth factor-1. It stimulates dental pulp stem cells, promotes odontogenic development and can therefore be used as a pulpotomy medication [97]. Kothari et al. evaluated CS and formocresol and reported a clinical success rate of 96.6% for primary-molar pulpotomy, with CS achieving a higher radiographic success rate (96.6%) compared to formocresol (89.6%) [98].

CS is a commonly used hemostatic agent, effective in controlling bleeding. Products like Celox (SAM Medical Products, Newport, OR, USA) utilize CS to control bleeding effectively in patients by forming a cross-linked barrier. In pulpotomy procedures, CS diluted with sterile saline is applied for 15–20 s to achieve hemostasis and support hard tissue formation [99].

- Pulpectomy

CS enhances the clinical functionality of biosynthetic materials like injectable hydrogels [100]. It acts as an effective carrier as demonstrated in experimental studies for antibacterial drugs, augmenting their efficacy, and due to its positively charged amino groups, it adheres well to mucosal surfaces or teeth, ensuring sustained contact during drug release [101]. CS can deliver a range of anticaries agents, such as metal ions, minerals, antibiotics, proteins, and deoxyribonucleic acid. However, its high hydrophilicity and positive charge can complicate the encapsulation and controlled release of cationic, hydrophobic, or large molecules [102]. CS-based drug delivery systems take advantage of their ability to adhere to oral tissues, facilitate mucosal delivery, and coat enamel or dentin. Additionally, CS can be incorporated into sealants, scaffolds, restoratives, adhesives, and resins. These properties are well utilized by some researchers to make CS-based obturating material, but all of this is restricted to in vitro studies.

- Regenerative

Progress in stem cell research has enabled in vitro and laboratory-based exploration of CS nanoparticles for stem cell-mediated repair, with a focus on regenerating the dentine–pulp complex and entire teeth. Experimental studies demonstrate CS as a promising material for guided tissue regeneration (GTR) due to its supportive properties for stem cell adhesion and proliferation [103]. Porous CS scaffolds enriched with growth factors and biomolecules promote odontoblast differentiation and biomineralization. CS-based scaffolds with signaling molecules and drugs enhance cell adhesion, differentiation, and proliferation. CS-sustained release systems, like thermosensitive hydrogels, are being explored for delivering vascular endothelial growth factors (VEGFs) to stimulate dental pulp stem cell proliferation and differentiation in vitro [97,104]. Despite these promising findings, translation to pediatric clinical practice remains unexplored due to the absence of well-designed clinical trials.

**Table 3:** Summary of studies on endodontic applications of chitosan in pediatric dentistry, along with material combinations of chitosan.

S. No	Endodontic Application	Author (Year)	Type of Study	Comparison Groups	Test Parameters	Result	Strength of Evidence
1	Intracanal medicament	Wassel M, 2023 [105]	In vitro	CS-CHX nanoparticles, CHX, double antibiotic paste (DAP), CS nanoparticles, CS-CHX nanoparticles, CHX, DAP CS nanoparticles, control	Direct and residual antimicrobial effect	CS-CHX nanoparticles demonstrated a faster and more potent effect than other groups.	Pre-clinical
2		Negin Farhadian, 2018 [106]	In vitro	CS, gelatin and calcium hydroxide.	Preparation, optimization and characterization	CS/Gelatin polymeric nano-CS showed sustained release of calcium ions.	Pre-clinical
3	Irrigant	Sarkees M, 2020 [107]	In vitro	17% ethylenediaminetetraacetic acid (EDTA), 0.2% CS, 10% trisodium citrate.	Smear layer removal efficacy	0.2% CS can act as an alternative to 17% EDTA.	Pre-clinical
4		Diatri Nari Ratih, 2020 [108]	In vitro	17% EDTA; 0.2% CS nanoparticles; 2.5% sodium hypochlorite.	Smear layer removal, microhardness and surface roughness of root canal	0.2% CS resulted in greater microhardness and decreased surface roughness of canal dentin.	Pre-clinical
5	Apexification	Flores-Arriaga, 2019 [109]	In vitro	CS dissolved in 1% or 2% acetic acid with the addition of calcium hydroxide or calcium chloride	Evaluate the sustained release of calcium ions, pH changes and cell viability	CS-based pastes exhibit sustained calcium ion release and in vitro cytocompatibility.	Pre-clinical
6	Regenerative	Belkadi Roumaissa, 2024 [110]	In vitro	CS nanoparticles, 0.2% CS irrigating solution, dual rinse, 17% EDTA, 10% citric acid, 2.5% sodium hypochlorite.	Dental Stem Cells SV40 (DSCS) cell viability and proliferation	CS nanoparticles and 0.2% CS irrigation solution were the least cytotoxic to DSCS. Dual Rinse^®^ and 10% Citric acid demonstrated significantly higher proliferation compared to non-TGF-β1-treated groups.	Pre-clinical
7		Wu S, 2019 [97]	In vitro	VEGF/CS/*β*-glycerophosphate (VEGF/CS/β-GP) hydrogel, CS/*β*-GP hydrogel	Effects of vascular endothelial growth factor	CS/β-GP hydrogel continuously releases VEGF and promotes odontogenic differentiation of dental pulp stem cells.	Pre-clinical
8	Pulp capping	Hengtrakool, 2023 [111]	In vitro	RMGICs, GI + epidermal growth factor (EGF), GI + C, GI + C (F) + EGF, GI + C(K) and GI + C(K) + EGF	Release of (EGF)	CS-modified RMGIC with added EGF prolongs the release of EGF and fluoride.	Pre-clinical
MATERIALS USED IN COMBINATION WITH CS							
1	Mineral Trioxide Aggregate (MTA)	Kamali A, (2017) [112]	In vitro	CS and zirconium oxide additives	Setting properties and biocompatibility	CS- and zirconia-containing cements had shortest setting time, highest strength, and enhanced apatite formation.	Pre-clinical
2		Mariyam M, 2023 [113]	In vitro	CS-MTA, Pro Root	Diametral tensile and compressive strength	CS-MTA showed highest strengths as well as strongest dentin–adhesive interface.	Pre-clinical
3	Biodentine	Abusrewil S, 2024 [114]	In vitro	Biodentine (BD), BD + CS	Physicomechanical and biological properties	CS-modified material significantly reduced human embryonic kidney (HEK 293) cell proliferation, indicating decreased biocompatibility.	Pre-clinical
4	Portland cement	Subhi H, 2021 [115]	In vitro	Accelerated Portland Cement (APC) and APC-CS	Effect on apoptosis, cell adhesion, dentinogenic/osteogenic differentiation and mineralization activity	APC-CS is nontoxic and enhances dentinogenic/osteogenic differentiation and mineralization of SHED, making it a promising alternative to calcium silicate-based cements for dentin/bone regeneration.	Pre-clinical

### 3.3. Summary

Based on the available literature on the uses of chitosan in the field of pediatric dentistry, the applications can be summarised by their evidence and clinical readiness level. While preventive applications of CS like mouthwashes, chewing gums and nanosilver fluoride formulations have early clinical studies and randomized controlled trials, other applications like restorative, regenerative, and endodontic applications remain at the preclinical stage (Table 4).

### 3.4. Future Research Perspectives

Despite the encouraging findings reported in preventive, restorative, and endodontic applications, it is important to recognize that the majority of available evidence originates from in vitro investigations, with comparatively few animal studies and limited clinical trials involving pediatric patients. Consequently, the translation of these findings into routine clinical practice remains challenging. Large, multicenter randomized controlled trials in children are needed to determine optimal concentrations, delivery forms, and long-term effectiveness for caries prevention, pulp therapy, and restorative applications. Emerging research areas include CS-based nanoparticle systems for targeted drug delivery, bioactive coatings for restorative materials and dental appliances, regenerative scaffolds for dentin–pulp complex regeneration, and smart biomaterials capable of controlled therapeutic agent release. In addition, future studies should investigate the effects of prolonged CS exposure on the developing oral microbiome. CS-based dentin replacement materials and bioadhesive polymers to improve shear bond strength and dentin bonding systems also need to be explored. At present, regenerative applications of CS should be regarded as experimental adjuncts rather than clinically established therapies, requiring rigorous animal studies followed by pediatric clinical trials to assess safety, efficacy, allergenicity and long-term outcomes both intra-orally and via systemic absorption. Addressing these gaps will enable CS to progress from a promising experimental biomaterial to a reliable component of pediatric dental care.

## 4. Conclusions

Although chitosan demonstrates considerable promise as a multifunctional biomaterial, the current evidence base remains largely dependent on in vitro investigations and limited clinical studies. Consequently, chitosan should presently be regarded as an experimental adjunct rather than a clinically established material for routine pediatric use. Future progress requires well-designed multicenter randomized controlled trials relevant to pediatric dental practice, formulation standardization, assessment of long-term clinical performance, and comprehensive safety evaluations before widespread clinical adoption can be recommended. The present review is limited by the predominance of in vitro studies, heterogeneity in chitosan formulations, variability in outcome measures, and the limited availability of pediatric clinical trials. Consequently, direct comparison among studies remains challenging. Also, although the antimicrobial activity of chitosan is generally considered advantageous for caries prevention and infection control, its broader effects on the developing oral microbiome remain insufficiently understood. Future studies should therefore evaluate not only reductions in cariogenic species but also the ecological effects of prolonged chitosan exposure on the pediatric oral microbiome using contemporary microbiome profiling techniques.

## Figures and Tables

**Table 4:** Summary table for applications of chitosan according to its current evidence level.

Application.	Evidence Level	Readiness Level
CS mouthwash	Clinical studies available	Early clinical application
CS chewing gum	Clinical studies available	Early clinical application
CS-Nanosilver fluoride	RCTs available	Early clinical evidence
CS based remineralization systems	In vitro	Pre-clinical
CS-GIC modification	In vitro	Pre-clinical
CS-Composite modification	In vitro	Pre-clinical
CS-based adhesive systems	In vitro	Pre-clinical
CS-based pulpotomy agent	Limited clinical studies	Early clinical evidence
CS intracanal medicament	In vitro	Pre-clinical
CS regenerative scaffolds	In vitro	Pre-clinical

## Data Availability

No new data were created or analyzed in this study. Data sharing is not applicable to this article.

## Supplementary Materials

The following supporting information can be downloaded at: https://www.mdpi.com/article/10.3390/ma19183951/s1, Table S1: Preferred Reporting Items for Systematic reviews and Meta-Analyses extension for Scoping Reviews (PRISMA-ScR) Checklist. Reference [116] is cited in the supplementary materials.

## Abbreviations

The following abbreviations are used in this manuscript:

MW	Molecular weight
kDa	kiloDaltons
CS	Chitosan gel
DD	Degree of deacetylation
*S. mutans*	*Streptococcus mutans*
ACP	Amorphous calcium phosphate
F	Fluoride
NSF	Nanosilver fluoride
CHX	Chlorhexidine
RCT	Randomized control trial
CS3H Lys	*N*-(2(2,6-diaminohexanamide)-Chitosan
Pchi	Phosphorylated CS nanoparticles
CB	Bioglass application on Chitosan
CBS	Chitosan/bioglass slurry
C/F	Chitosan/fluoride microparticles
PFS	Pit and fissure sealant
GIC	Glass ionomer cement
FTIR	Fourier Transform Infrared Spectroscopy
RMGIC	Resin-modified glass ionomer cement
GTR	Guided tissue regeneration
VEGF	Vascular endothelial growth factor
DAP	Double antibiotic paste
EDTA	Ethylenediaminetetraacetic acid
DSCS	Dental Stem Cells SV40
VEGF/S/β-GP	Vascular Endothelial Growth Factor/Chitosan/*β*-glycerophosphate
EGF	Epidermal growth factor
MTA	Mineral Trioxide Aggregate
BD	Biodentine
HEK 293	Human embryonic kidney cells
APC	Accelerated Portland Cement
